# A Multi-scale Biophysical Approach to Develop Structure-Property Relationships in Oral Biofilms

**DOI:** 10.1038/s41598-018-23798-1

**Published:** 2018-04-09

**Authors:** J. Pattem, M. Davrandi, S. Aguayo, E. Allan, D. Spratt, L. Bozec

**Affiliations:** 1 Biomaterials and Tissue Engineering, Eastman Dental Institute, University College London, London, UK; 2 Microbial Diseases, Eastman Dental Institute, University College London, London, UK

## Abstract

Over the last 5–10 years, optical coherence tomography (OCT) and atomic force microscopy (AFM) have been individually applied to monitor the morphological and mechanical properties of various single-species biofilms respectively. This investigation looked to combine OCT and AFM as a multi-scale approach to understand the role sucrose concentration and age play in the morphological and mechanical properties of oral, microcosm biofilms, *in-vitro*. Biofilms with low (0.1% w/v) and high (5% w/v) sucrose concentrations were grown on hydroxyapatite (HAP) discs from pooled human saliva and incubated for 3 and 5 days. Distinct mesoscale features of biofilms such as regions of low and high extracellular polymeric substances (EPS) were identified through observations made by OCT. Mechanical analysis revealed increasing sucrose concentration decreased Young’s modulus and increased cantilever adhesion (p < 0.0001), relative to the biofilm. Increasing age was found to decrease adhesion only (p < 0.0001). This was due to mechanical interactions between the indenter and the biofilm increasing as a function of increased EPS content, due to increasing sucrose. An expected decrease in EPS cantilever contact decreased adhesion due to bacteria proliferation with biofilm age. The application OCT and AFM revealed new structure-property relationships in oral biofilms, unattainable if the techniques were used independently.

## Introduction

Oral biofilms are complex microbial communities^1^, embedded in a matrix of extracellular polymeric substances (EPS)^2,3^. EPS accounts for up to 90% of a biofilms’ total mass^4,5^, consisting of polymers, mainly extracellular DNA (eDNA), polysaccharides, proteins and lipids of bacterial and salivary origin^2,6^. Its’ role is to provide a protective sheath^7^, encapsulating the multi-species bacterial complex, proliferating into distinct morphotypes, such as bulbous micro-colonies, maintaining its structural integrity^7^. Oral biofilm formation is a hierarchical process^8^, consisting of surface attachment by primary bacterial colonizers, such as streptococci^9^. This is followed by subsequent attachment of secondary, later and bridging colonizers, such as Actinomyces, Veillonella and fusobacterium respectively^8,9^.

A biofilms hierarchical nature^8^ is an important feature to consider when contemplating how to analysis of their morphological and mechanical properties. During formation and proliferation, a biofilms’ structural range extends from single molecular EPS constituents to bulbous micro-colonies and finally, fully formed mesoscale surface coverings^4^. Applying techniques that span a range of length scales, are capable of *in-vitro* analysis and preserve biofilm structural and mechanical integrity are vital in the development of structure-property relationships.

Traditionally, binding assays such as fluorescence staining have enabled researchers to extensively investigate a biofilms microbiology with increasing detail since the 1980′s^10,11^. Since then, characterizing this multi-layered and multi-colonial bacterial community has led to the biological mapping of this complex, hierarchical structure^12^. From a structural perspective, common characterization methods include scanning electron microscopy (SEM)^13–15^ and confocal laser scanning microscopy (CLSM)^15,16^. These techniques only provide basic morphology and quantitative live – dead ratios respectively^16^. While these techniques can yield 3D structural information, they can be damaging to the specimen^17^ and do not provide any mechanical information.

Over the last 5 years, investigators have increasingly utilized atomic force microscopy (AFM) to obtain mechanical information from a variety of single-species biofilms, under several *in-vitro* conditions^18–20^. Through non-destructive indentation, low applied force and known indenter geometry, mechanical properties such as elastic modulus, Young’s modulus and adhesion can be obtained from generated force-displacement curves^21^. Advances in AFM technology have allowed users to perform indentations on a specimen in a point-wise array, generating both morphological and mechanical properties simultaneously, termed, force-volume imaging (FVI)^22^. It has been used to determine the morpho-mechanical profiles of single bacterial species and cells with success^23,24^.

To non-destructively observe biofilm structure, investigators have increasingly utilized optical coherence tomography (OCT)^25–27^. OCT is a versatile tool for medical imaging and has been used to resolve 2D and 3D images of biological structures since its invention in 1991^26–28^. OCT provides depth-resolved analysis of backscattered light via an interferometer^27^ using a low-coherence light source, such as a near-infrared probe beam. This is traversed across a surface of interest and is possible to resolve image depths of several centimeters, with a lateral and axial pixel resolution of <5 µm^26,27^. OCT provides non-invasive, label-free and real-time *in-vitro* characterization of biofilms. Scattering or grey-level intensity profiles can be produced^29^ enabling certain aspects of biofilms such as voids and microcolonies to be determined with respect to depth^26^. The application of OCT to biofilms is not new. OCT’s imaging and scattering intensity capabilities have been applied to wide range of fields from waste-water treatment systems^27^, biofouling in bioreactors^27^ to *ex-vivo* oral biofilms^30^. Investigators have focussed applying OCT on mechanisms of biofilm attachment^26^ and detachment under various flow conditions^25,27^ and on a variety of surfaces^30^. OCT has enabled mixed species biofilm mass to be quantified on differing dental materials during growth^30^. It has also been used to assess biofilm removal strategies such as chlorohexidine, revealing a collapse of biofilm structure in semi-real-time^31^.

While OCT and AFM have been individually applied to monitor the morphological and mechanical properties of single species biofilms respectively, there have been no reports on using OCT and AFM in a combinatorial approach on proliferated, multi-species biofilms. With investigators focussing on the microbiological changes of biofilms with regard to sucrose and age^6,32,33^, nothing has been reported on what these factors play in their biophysical characteristics. This is of particular interest to the dental community, producing biofilms on mineralized surfaces as reflected by dental plaque in the oral cavity^6^.

The aim of this investigation is to apply OCT and AFM to determine the meso- and micro-scale morphological and nano-mechanical properties of oral biofilms under physiological conditions. Furthermore, to determine if this approach can be used to develop structure-property relationships in multi-species biofilms, with regard to increasing sucrose and age.

## Materials and Methods

### Hydroxyapatite substrates

Nine, 5 mm diameter HAP discs were fabricated from <75 µm particle size HAP (Sigma-Aldrich, UK) using a pressing die under 2 Tonne (George E. Moore & Sons Ltd, UK). These were randomly assigned into 2 groups, with group 1 containing 5 specimens and group 2 containing 4. Group 1 specimens were biofilm-free and were used to monitor HAP surface morphology. HAP disc morphology was analyzed using average roughness (R_a_) for normality (p > 0.05). Group 2 specimens were used to monitor biofilms using methods described below.

### Biofilm Formation

Microcosm biofilms were grown on group 2 HAP discs using a feed batch culture approach. Sterilized HAP discs were placed horizontally in a 96-well plate (Nunc Nunclon Delta). Biofilms were formed on HAP discs from stimulated, pooled human saliva (n = 15) using two different growth media previously termed nutrient poor and nutrient rich.

These are composed of the following. Nutrient poor (NP) was artificial saliva based, containing 1 g/L of lab-lemco, 2 g/L of yeast extract, 5 g/L of protease peptone, 2.5 g/L of mucin from porcine stomach (Type III), 0.35 g/L of sodium chloride, 0.2 g/L of calcium chloride and 0.2 g/L potassium chloride. After autoclaving, 1.25 mL of filter sterilized (0.22 *µ*m) 40% (w/v) urea solution and 2 mL of filter sterilized (0.22 *µ*m) 50% (w/v) sucrose solution were added per litre. Nutrient rich (NR) was based on Brain Hearth Infusion (BHI) containing 37 g/L of BHI powder and 2.5 g/L of mucin from porcine stomach (Type III) in 900 mL dH_2_O. After autoclaving, 100 mL of filter sterilized (0.22 *µ*m) 50% (w/v) sucrose solution was added. All materials were sourced from Sigma-Aldrich, UK.

Pooled human saliva (1 ml) was inoculated in 7 ml of NP with 0.1% (w/v) sucrose and NR with 5% (w/v) sucrose. 180 µL aliquots of both inoculum were added to the wells and incubated at 37 °C in 5% CO_2_, for a total of 120 h. Each growth media was replaced by pipetting at 24 h intervals. Biofilm specimens grown in either nutrient poor or nutrient rich conditions were collected at 72 h (D3) and 120 h (D5) for analysis.

### Optical Coherence Tomography

VivoSight Multi-Beam Swept Source OCT system (Michelson Diagnostics Ltd, UK) was used to observe the cross-sectional morphology of each biofilm covered HAP disc, including 1 HAP biofilm free surface. This system uses a class I laser (λ = 1305 nm) and scans at a rate of 10 kHz. The default scanning volume was set to 6 × 6 mm and approximately 2 mm deep. For each samples, a total of 500 B-scans were recorded over that volume. Each B-scan was recorded 10 µm apart and with a pixel size of 4.53 µm. Specimens were attached to a 35 mm petri dish (Thermofisher, UK) using perfluoropolyether lubricant (Fomblin, UK) and submerged in phosphate buffered saline (PBS) (Lonza Biowhittiker, UK) for 1 hour before analysis.

### AFM Imaging

A JPK Nanowizard 1 AFM (JPK Instruments Ltd, Germany) was used to obtain example 50 × 50 µm, 10 × 10 µm and 3 × 3 µm AFM images from each biofilm covered HAP disc under PBS conditions. These were conducted using MSNL-10 cantilevers (Bruker Ltd, France) after 1 hour in PBS.

### AFM Probe modification and Force-Volume Imaging

A JPK Nanowizard 1 AFM (JPK Instruments Ltd, Berlin) was used to functionalize NPO-10 tip-less cantilevers (Bruker Ltd, France). Cantilevers were modified with 10 µm borosilicate spheres (Whitehouse Scientific, UK) using a UV curing resin (Loctite, UK). Successfully glass sphere attached cantilevers were cured under UV light (λ = 400 nm) for 5 minutes. Successfully functionalized cantilevers were calibrated before analysis generating a spring constant of 0.36 ± 0.18 N/m. Imaging and mechanical analysis was performed by AFM operating in force-volume imaging (FVI) mode.

AFM FVI was used to monitor 2.5 × 2.5 µm areas of each biofilm covered HAP disc under PBS conditions. These were conducted using 10 µm borosilicate bead functionalized NPO-10 cantilevers to determine the mechanical properties of individual bacteria and EPS after 1 hour in PBS.

AFM FVI was performed on at total of 9 separate areas of 80 × 80 µm, on each biofilm covered disc, at least 2 mm apart, at a resolution of 16 × 16. Consequently, only 3 separate areas were successful generating 768 indentations per sub-group, across the total biofilm covered HAP disc. FVI’s were conducted on specimens after 1 hour PBS hydration and monitored under PBS hydrated conditions. 80 × 80 µm force volume images were randomly selected from the 3 successful FVI’s and a normalised coloured scale bar was applied.

Young’s modulus (kPa) and adhesion (N) were obtained from individual force distance curves, using dedicated software (JPK Instruments, Ltd). Young’s modulus of specimens were extracted using the Hertz model^34^ for a spherical indenter, where force is related to the indentation depth from the Equations 1, 2 and 3

1 $$F=\frac{E}{1-\nu .\nu }[\frac{a.a+R.R}{2}\,\mathrm{ln}\,\frac{R+a}{R-a}-aR]$$

2 $$\partial =\frac{a}{2}\,\mathrm{ln}\,\frac{R+a}{R-a}$$

3 $$a=\surd R\partial $$

where, R is the indenter radius, E is Young’s modulus, ∂ is indenter depth, v is Poisson’s ratio which is 0.5^35^. The Hertz model is used as it has been extensively applied to biofilm mechanical analysis using AFM^18–20^. Adhesion force was obtained from the deflection distance of the cantilever and the cantilever spring constant using Equation 4,

4 $$F=k.{\rm{\Delta }}L$$

where, *F* is force in nN, *k* is the cantilever spring constant and *ΔL* is the deflection distance in nm^36^.

### OCT Image Extraction and Analysis

Selected B-scans were chosen randomly from each 500 B-scan image stack. Grey intensity profiling was conducted by extracting a vertical scattering profile (A-scan) using ImageJ software^37^.

### AFM Data Extraction, Statistics, and Analysis

Morphological analysis was conducted on 5 biofilm-free HAP discs monitoring two different 80 × 80 µm areas per sample using average roughness (R_a_). The analysis was conducted using dedicated software to determine if they were normally distribute using a Shapiro-Wilk normality test. Biofilm covered HAP disc Young’s modulus (kPa) and adhesion (nN) were obtained from each sucrose and age subgroup using dedicated software (JPK Instruments Ltd, Germany). Mechanical data were tested for normality using a Shapiro-Wilk test and significant differences were analyzed using a series of Kruskal-Wallis 1-Way ANOVAs, with a Dunn’s multiple comparison procedures in terms of sucrose concentration and age at p < 0.0001.

## Results and Discussion

### Mesoscale Morphological Characterization

**Figure 1:**
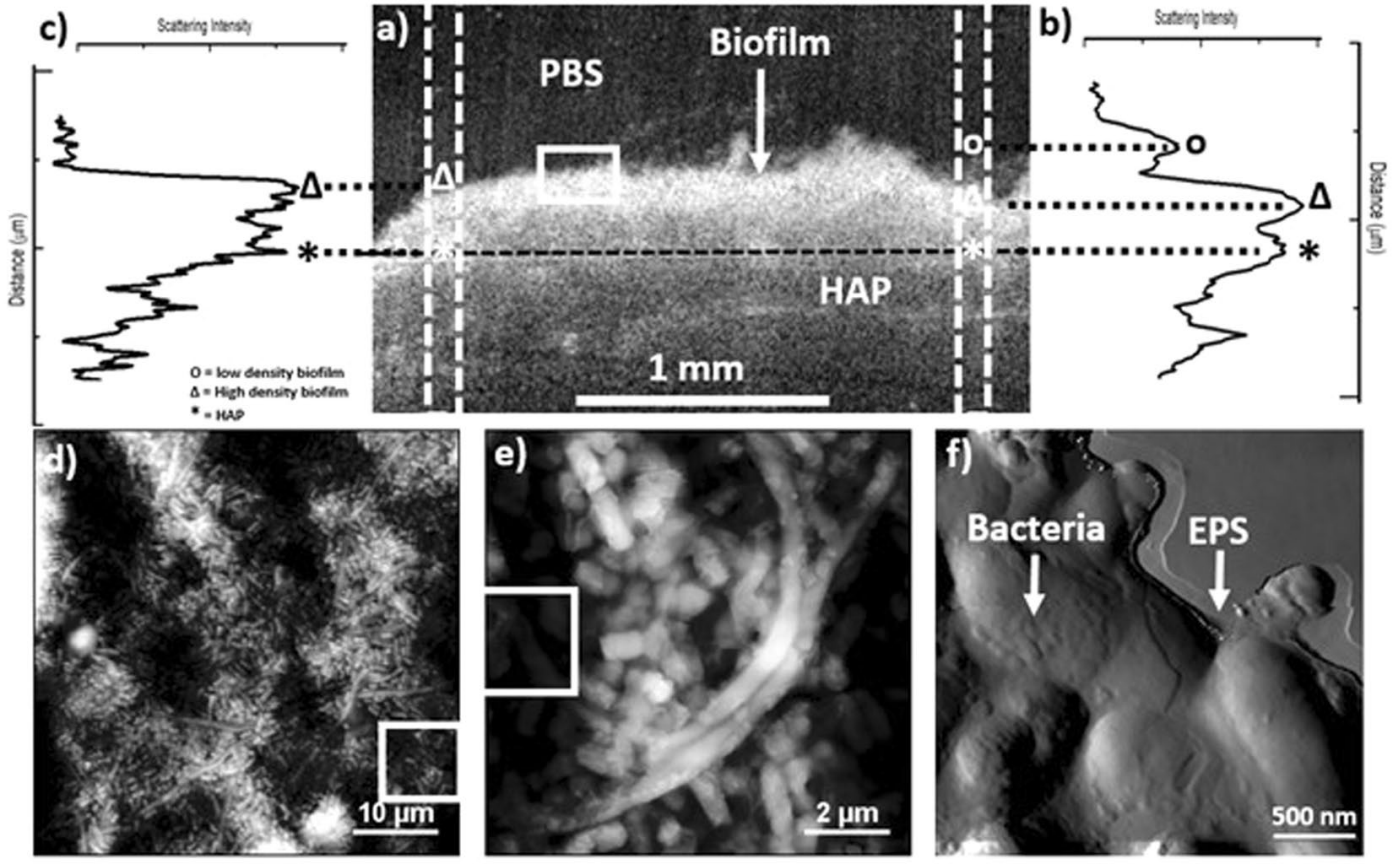
Showing (**a**) a typical OCT cross-section slice from NR-D5, (**b**) the corresponding vertical scattering profile selected from the right-hand dashed region of (**a**), (**c**) the corresponding vertical scattering profile selected from the left-hand dashed region of **(a**). (**d**) shows a 50 × 50 µm image of the boxed area on the NR-D5 biofilm in (**a**), (**e**) a 10 × 10 µm area selected from the boxed bottom right corner of (**d**) and (**f**) a 3 × 3 µm image from the left-hand boxed area of (**e**).

OCT imaging and scattering intensity profiling were applied to biofilms cultivated on HAP discs with increasing sucrose concentration and age, under static PBS conditions. Biofilm specimens were analyzed at room temperature (22 °C) on the day of extraction from the well plate. Figure 1(a) shows a typical OCT cross-sectional B-scan of a nutrient-rich, day 5 biofilm (NRD5) on a HAP disc. The biofilm exhibits a heterogeneous morphology, characteristic of *in-vitro* and *ex-vivo* oral biofilms^30^. Figure 1(b) and (c) shows the corresponding signal intensity from selected dashed regions on Fig. 1(a). This is the intensity of light back-scattered from the sample^27^. Areas of high-density backscatter more light than those of low density^27^. Areas of varying density are exhibited by the grey scale in the image. Low and high-density regions in Fig. 1(b) denoted by (o) and (Δ) respectively are visually different, indicating differences in the biofilms bacterial density^19,20^. Conversely, in Fig. 1(c), there is only a high-density region (Δ), further highlighting a biofilms morphological heterogeneity^30^. Considering the limitations of OCT, areas of low visual scattering do not necessarily represent unapparent biofilm structure. Here, we hypothesize that areas of low optical density have a high EPS to bacteria ratio, while areas of low density have a high bacteria to EPS ratio. The corresponding vertical scattering intensity profile in Fig. 1(b) is characterized by a high EPS to bacteria ratio region with low scattering intensity (o). This is then followed by a high bacteria to EPS ratio region with greater scattering intensity (Δ). Taking into consideration the heterogeneous cross-sectional morphology of biofilms, it is possible to differentiate regions of high and low EPS to bacteria ratio regions across the surface using OCT and scattering intensity profiles at the mesoscale^27,30^.

**Figure 2:**
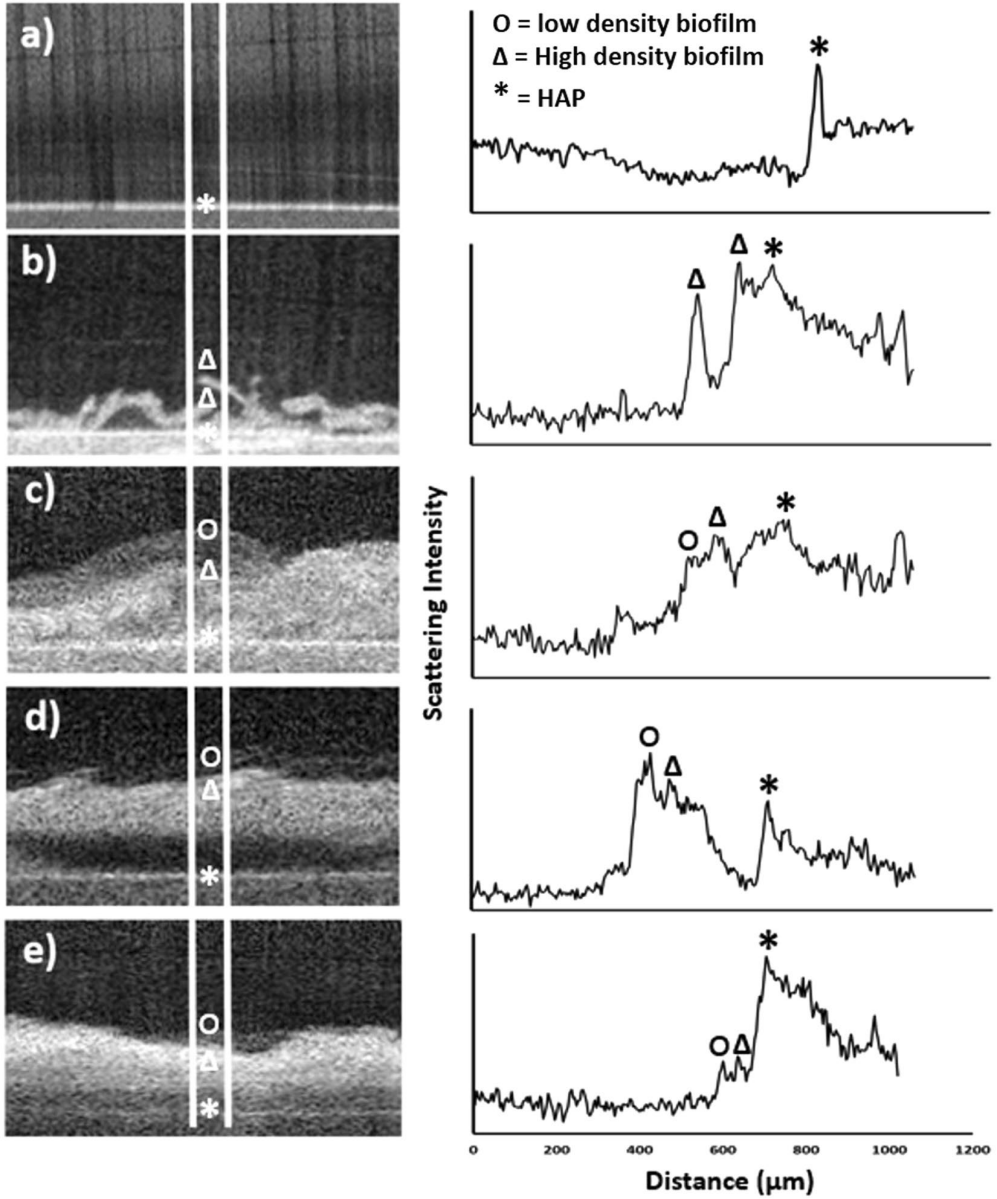
Showing left OCT imaging and right, corresponding scattering intensity profiles of (**a**) control HAP, (**b**) NPD3, (**c**) NPD5, (**d**) NRD3 and (**e**) NRD5. White lines on left show area selected for scattering intensity profiling. Scattering intensity graphs are highlighted with locations of * (hydroxyapatite), Δ (high density biofilm) and ο (low density biofilm).

Figure 2(a–e) shows selected B-scans of HAP, NP and NR cultured biofilms at 3 and 5 days of age respectively. Their corresponding scattering intensity profiles are also shown with variations in bacterial density. Figure 2(a) of an HAP disc shows a peak intensity in the scattering profile at the surface of HAP, as reflected in Fig. 1(a) and (b). Nutrient-poor biofilms shown in Fig. 2(b,c) exhibit an increase in biofilm deposition with age^26^, characterized by the cross-sectional B-scan. Figure 2(b) of nutrient-poor day 3 biofilm exhibits low growth, with interstitial spacing between clusters of bacteria on the HAP substrate. Variations in the bacterial density of nutrient-poor day 5 in Fig. 2(c) can also be observed. A high EPS to bacteria ratio region is situated directly above a high bacteria to EPS ratio region. Nutrient-rich biofilms at 3 and 5 days age shown in Fig. 2(d) and (e) exhibit similar morphology, with some lifting occurring at 3 days in Fig. 2(d). Intensity variation, such as low and high EPS to bacteria ratio regions can be observed in the corresponding intensity profiles. It is clear from Fig. 2(a–e) that OCT provides utility in monitoring biofilms at the mesoscale level, identifying bacterial deposition and structural variance with regard to changes in sucrose concentration and age.

Other complementary techniques such as confocal laser scanning microscopy (CLSM)^25,26^ and scanning electron microscopy (SEM)^30^ have been used to monitor biofilms. These have been utilized to resolve other features such as individual bacteria and EPS at smaller length scales^38,39^. These techniques can be destructive to the biofilm through staining and fixing and may affect their mechanical integrity. One ought to characterize smaller-scale features such as individual bacteria and EPS using AFM. This also provides a non-destructive approach, capable of analysis under physiological conditions, with no sample preparation.

Biofilms are by their very nature are heterogeneous as a function of surface location and depth^40^. Cross-sectional analysis of surface and subsurface features by OCT can provide valuable information on where to land an AFM probe for effective analysis. As AFM image formation and mechanical analysis are conducted blind, it can be very difficult and time-consuming to locate appropriate areas. For example, Fig. 2(b) shows biofilm areas partially detached from the surface. Accessing any morpho-mechanical information on these areas of high heterogeneity will be extremely difficult when using AFM. Allowing OCT to provide a snapshot cross-sectional view of biofilms assists effective AFM topographical and mechanical analysis.

### Nanoscale Morphological Characterisation

AFM is a powerful tool to image biofilms and bacteria under physiological conditions, at the micro to nanoscale^41–43^. Figure 1(d) shows a typical 50 × 50 µm image of a nutrient-rich biofilm at 3 days of age. At this scale, bacteria can be identified across the entire analyzed surface. The biofilm exhibits a heterogeneous morphology with varying heights denoted by the grey scale. Figure 1(e) shows a 10 × 10 µm taken from the lower right-hand region of Fig. 1(d). Individual bacteria can be identified due to their different morphological characteristics. It is clear that the biofilm is multispecies, for example, streptococci and Fusobacterium can be recognized due to their short and elongated morphology respectively^44^. Figure 1(f) shows a 3 × 3 µm Z-drive image taken from the left-hand side of Fig. 1(e). Bacteria are observable, covered with a layer EPS, which is also prominent at the periphery, encapsulating the biofilm^4^. The application of AFM to determine the morphological nature of varying biofilms has provided utility in resolving surface features at the single bacteria and composite level^45–47^. Topographical AFM imaging has enabled researchers to resolve the surface features of individual bacterial species^45–47^, including their characteristics relative to changes in the environment^47^. AFM provides access to the biophysical properties of individual species. It is a significant competitor to other techniques in its ability to resolve bacteria and biofilm features, under physiologically relevant conditions.

**Figure 3:**
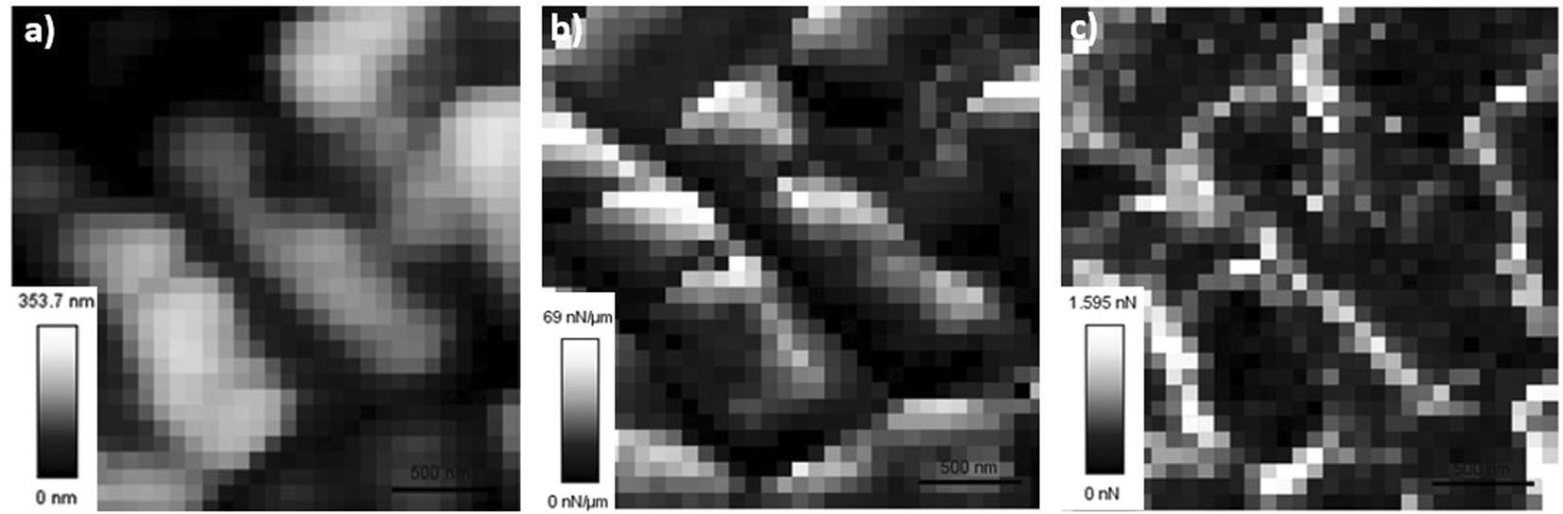
Showing 3 × 3 µm AFM FVI maps of (**a**) topography, (**b**) elastic modulus (nN/µm) and (**c**) adhesion (nN) of a nutrient rich day 3 biofilm.

AFM imaging in FVI mode was applied to biofilms cultivated on HAP discs with increasing sucrose concentration and age, under static PBS conditions. Biofilm specimens were analyzed at room temperature (22 °C) on the day they were extracted from the well plate. FVI is a point-wise force-curved based imaging method, generating a force curve at each pixel from a chosen resolution^23,48^. Height images are extracted from the maximum indentation force in each force curve^21,23^, generating a topographic height map. Variations in height (cantilever movement) are an indicator of morphological heterogeneity at the microscale. Figure 3(a) shows an example 3 × 3 µm FVI height image of a nutrient-rich day 3 biofilm under physiological conditions. Numerous bacteria are clearly visible, due to their protrusion from the surface, denoted by the grey-scale.

**Figure 4:**
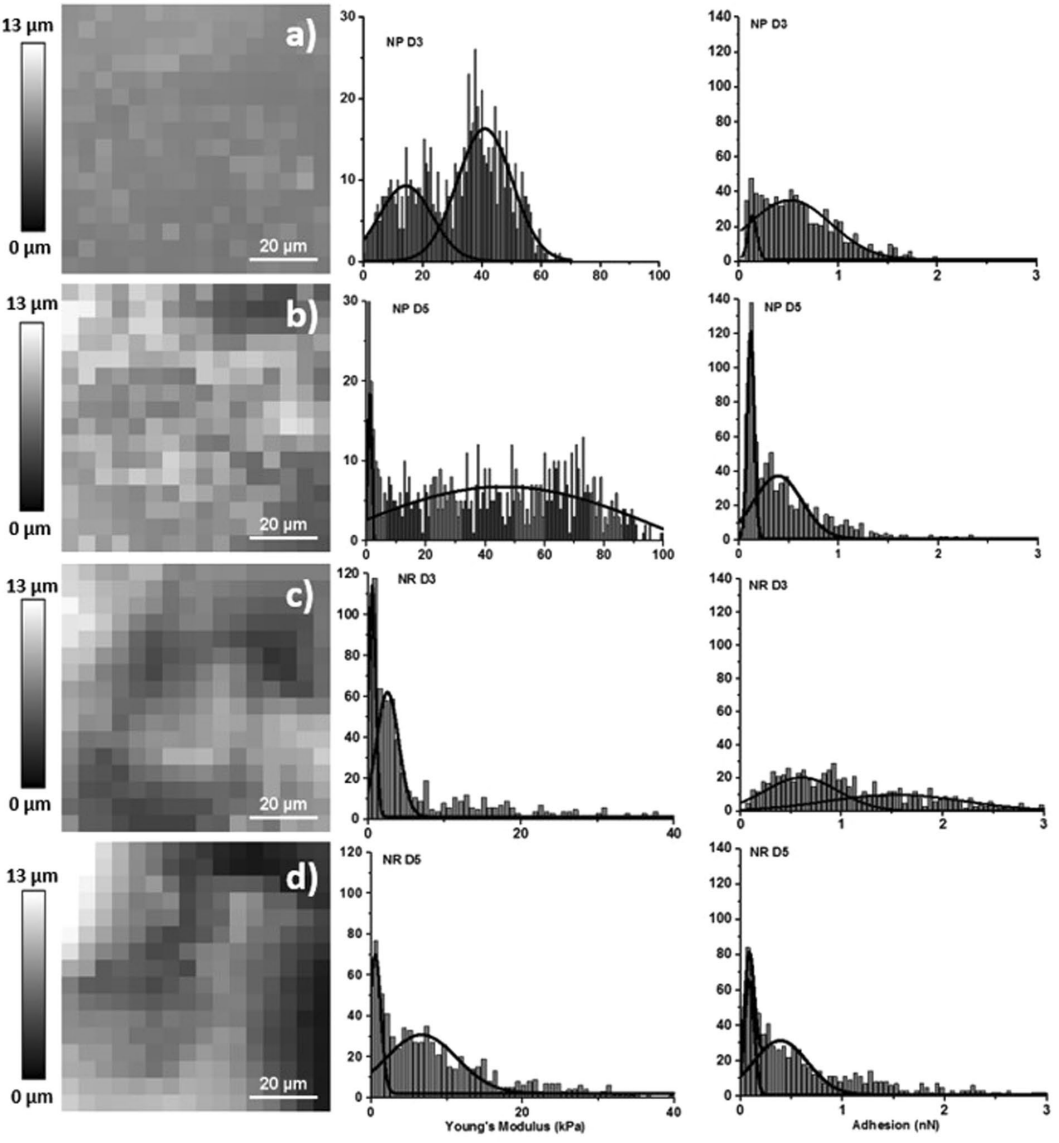
Showing example 80 × 80 µm FVI Images at a resolution of 16 × 16 with their corresponding Young’s modulus and adhesion histograms of (**a**) NP D3, (**b**) NP D5, (**c**) NR D3 and (**d**) NR D5.

Example normalized FVI height maps of nutrient-poor and nutrient-rich biofilms at 3 and 5 days of age are shown in Fig. 4(a–d) respectively. The cantilever heights during non-destructive contact are shown to increase, moving from 3 to 5 days of age, in both nutrient poor and nutrient-rich biofilms, shown in Fig. 4(a,b) and (c,d) respectively. This shows that an increase in both age and sucrose concentration produces biofilms with a more heterogeneous morphological profile at this scale, like those found in OCT imaging at the mesoscale. Investigators have focussed on the topography of varying single-species biofilms, showing variations in profile heights and roughness^43^. Here, these were found to change under varying culture conditions.

#### Nanomechanical Properties of Biofilms: Young’s Modulus

The advantage of AFM FVI is its capability to both image and obtain biofilm mechanical properties of a specimen simultaneously^48^. Corresponding elastic modulus maps for Fig. 3(a) are shown in Fig. 3(b). Elastic modulus is obtained from the slope of the retract curve during its phase shift and is defined as the resistance to indentation by an applied force^21^. Applying contact mechanics models such as Hertz, Young’s modulus, a measure of specimen elasticity^21^ can be obtained.

Mechanical variation with respect to indentation location^23^ is apparent in Fig. 3(b), shown by the elastic modulus grey scale. Indentation occurring on the bacteria surface show higher elastic modulus compared to those between bacteria. As EPS is secreted from bacteria, at this level it is assumed it will be most prominent between opposing bacteria, as shown in Fig. 1(e). Figure 3(b) shows that EPS is lower in elastic modulus compared to bacteria.

Young’s modulus histograms of nutrient-poor and rich at 3 and 5 days of age are shown in Fig. 4(a–d). These were taken by combining all data collected from 3 areas at least 2 mm apart on each disc, at a resolution of 16 × 16, generating n = 768 individual force-distance curves. Increasing sucrose concentration was shown to significantly decrease Young’s modulus at both stages of age (p < 0.0001). Binomial distributions are apparent in the histograms, indicating contact mechanics variations between the indenter and low and high bacteria density regions respectively. Nutrient-poor and rich at day 3 decreased in Young’s modulus from between 14.35 ± 1.75–41.05 ± 0.97 kPa to 0.55 ± 0.02–2.57 ± 0.17 kPa (P < 0.0001), shown in Fig. 4(a) and (c) respectively shown in Table 1. At day 5, Young’s modulus decreased from between 1.17 ± 0.08–46.07 ± 1.87 kPa to 0.56 ± 0.06–6.66 ± 0.46 kPa (p < 0.0001), shown in Fig. 4(b) and (d) and Table 1 respectively. There was no significant difference in Young’s modulus between each age within nutrient poor and nutrient-rich groups, exhibiting p values of p = 0.8678 and p = 0.0028 respectively. Young’s modulus values obtained here coincide with previously reported data on single species biofilms^18–20,49,50^. Mixed species biofilms with high sucrose concentrations studied here reflect similar mechanical properties to single species biofilms under similar conditions^18,19,49,50^. This maybe a consequence of streptococci dominating the bacterial community with the introduction of sucrose^51^. Also, biofilms were produced under aerobic conditions but in the presence of CO_2_, reflecting the *in-vivo* environment. As the biofilm matures, the proportion of obligate anaerobic species may have increased, reflecting similar mechanical properties to those found in single-species biofilms.

**Table 1:** Showing the 1^st^ and second Young’s modulus (kPa) and adhesion (N) distributions of NP and NR biofilms at 3 and 5 days age.

Distribution	Young’s Modulus (kPa)	Young’s Modulus (kPa)	Adhesion (nN)	Adhesion (nN)
	1st	2nd	1st	2nd
NP D3	14.35 ± 1.75	41.05 ± 0.97	0.13 ± 0.01	0.51 ± 0.04
NP D5	1.17 ± 0.08	46.07 ± 1.87	0.12 ± 0.00	0.39 ± 0.02
NR D3	0.55 ± 0.02	2.57 ± 0.17	0.60 ± 0.02	1.5 ± 0.16
NR D5	0.56 ± 0.06	6.66 ± 0.46	0.08 ± 0.00	0.39 ± 0.02

Nutrient poor biofilms exhibited secondary distributions in Young’s modulus of 41.05 ± 0.97 kPa and 46.07 ± 1.87 kPa respectively shown in Table 1. Increasing sucrose concentration reduced the Young’s modulus to 2.57 ± 0.17 kPa and 6.66 ± 0.46 kPa respectively shown in Table 1. A decrease in Young’s modulus associated with an increase in sucrose concentration can be explained by an increase in the bacteria’s production of EPS^52,53^. As sucrose is broken down by oral bacteria, it acts as a substrate for the synthesis of EPS by glucosyltransferases (GTFs)^54^. Consequently, increasing the EPS content will impact the contact mechanics variations occurring between the AFM cantilever and the biofilm under investigation. As bacteria are more rigid structures than EPS^55^, also shown in Fig. 3(b), low sucrose culture conditions are expected to have more EPS-free bacteria interacting with the cantilever. This results in higher composite Young’s modulus, evident in Young’s modulus histograms of nutrient-poor day 3 and 5 in Fig. 3(a) and (b) respectively.

#### Nanomechanical Properties of Biofilms: Adhesion

Corresponding adhesion maps for Fig. 3(a) are shown in Fig. 3(c). Adhesion, defined as the force required to remove the cantilever from the surface^21^, is obtained from the pull off region of the retract curve^21,56^. As in the elastic modulus map shown in Fig. 3(b), adhesive variation with respect to indentation location is apparent, shown in Fig. 3(c). Figure 3(c) shows higher adhesion between bacteria compared to the bacteria surface, indicative of EPS. Adhesion analysis was applied to the same force curves as in Young’s modulus calculations. Increasing sucrose concentration increased overall cantilever adhesion between nutrient poor and nutrient-rich groups at both 3 and 5 days (p < 0.0001). Table 1 shows biofilm adhesion increased with increasing sucrose (p < 0.0001). Increasing age was shown to decrease adhesion within the nutrient-poor group at 5 days shown in Table 1. This was also found to decrease in the nutrient rich group at 5 days (p < 0.0001).

Adhesion values obtained here coincide with previously reported data on singles species bacteria^36,49,50,56^. One study used sharp indenters and monitored the specimen in the air, leading to a reduction in the EPS water content and increased density of constituents. Other studies have obtained similar mechanical properties for both early stage and mature biofilms^49^. That particular investigation obtained significant differences in adhesion between differing strains of P. *aueruginosa*. While maturation was a key factor^49^, the nutrient composition did not differ and only 10 force plots were analysed per group.

Increasing sucrose concentration increased biofilm adhesion, while age decreased it. An increase in adhesion with increasing sucrose concentration can be associated with the increased production of EPS interacting with the cantilever. As a biofilms mechanical properties are dominated by EPS^57^, it can be assumed the superficial adhesion of a biofilm to the AFM cantilever is also dominated by EPS. Increased EPS – cantilever contact increased the force required to remove the cantilever from the surface shown in Table 1. The effect of age decreasing adhesion may be associated with bacteria proliferation in the biofilm. Increased bacteria content i.e increased bacterial density will increase the interactions between the indenter and bacteria. Therefore, reducing the pull of forces as fewer EPS interactions on the cantilever are occurring.

### Merits of a Multi-scale Analysis Approach

A biofilms hierarchical nature requires analysis techniques that complement its structural properties, spanning from the single bacteria to mesoscale level. These techniques must be non-destructive and allow for analysis under *in-vitro* physiological conditions. This is vital when developing structure-property relationships in biofilms as their formation, proliferation, and maturation span this large range^2–6^.

Here, OCT revealed side on views of biofilms at the mesoscale allowing depth analysis, unattainable by AFM. While nutrient-poor biofilms exhibited differences in morphology, nutrient-rich were morphologically similar. AFM’s ability to complement morphology with micro to nanoscale mechanical properties revealed differences in contact mechanics occurring between the indenter, bacteria, and EPS, unattainable by OCT. These were found to change as a function of the biofilms culture conditions.

It must be noted that an average of 9 areas were monitored per specimen for successful mechanical analysis. In terms of elucidating mechanical properties from biofilms, the ability to land a modified cantilever onto its surface is extremely difficult, especially under physiological conditions. This is due to the biofilms morphological heterogeneity^58^ and extremely soft ultra-structure^18–20^, particularly in low bacteria density and high EPS content regions. Meaning, very small indentation forces and high cantilever z-lengths are required when moving from one indentation to another. Low applied forces (~6 nN) used in this investigation enabled the mechanical response to be solely that of the biofilm surface, as large forces would have indented below the contact point, resulting in disruption of the structure. Not only this, monitoring large 80 × 80 µm areas as conducted here over-shadowed the mechanical effect of biofilm constituents such as bacterial species, providing a composite average value. The use of low indentation forces and large spherical tips enabled non-destructive probing of each biofilm. Monitoring relatively large analysis areas enabled the elastic response to be representative of the bulk structure.

Only the structural morphology and mechanical properties of biofilms were the focus of this study, while bacterial taxa, their spatial distribution and EPS content were not. Future studies may wish to use already well-characterized biofilms such as those developed using the Zurich biofilm model^59^. This may enable their morpho-mechanical response to be associated with potential markers in bacterial population. Techniques such as fluorescence *in-situ* hybridization (FISH) and CLSM have been used to characterize the spatial distribution of bacteria in oral biofilms, targeting specific bacteria via 16S rRNA sequences^60^. Furthermore, as EPS is the principal determinant in the mechanical response of biofilms^57^, identifying shifts in EPS chemistry may identify chemical markers changing the overall biophysical properties of biofilms. Although, to determine their morphological and mechanical response to the chemical changes at the composite level, this must be conducted either separately, using a separate set of specimens, or under *in-vitro* conditions. Techniques using specimen staining can be damaging to the biofilm^17^, while others destroy the specimen during preparation such as transmission electron microscopy (TEM)^61^.

The aim of this investigation was to determine if OCT and AFM could be used as a multi-scale approach to develop structure-property relationships in oral biofilms, *in-vitro*. This was successfully applied and was able to monitor a significant morphological and mechanical response as a function of biofilm culture conditions. These techniques and applied methodology can now be used in the future to determine and further existing structure-property relationships in biofilm growth, proliferation, and maturation. This is the first study of its kind into developing a non-destructive, multi-scale approach and is not restricted to the type of biofilm under investigation, nor the *in-vitro* physiological conditions in which researchers wish to investigate. This approach can be applied to a range of biofilms types such as those found in waste-water treatment systems, bio-reactors, catheters and those found in the oral cavity. Varying physiological conditions can also be applied to reflect specific environments, which include chemical strategies to remove biofilms, even under flow.

## Conclusion

Developing effective characterization methods to nondestructively analyze multispecies biofilms *in-vitro* is a step forward in identifying structure-property relationships. While the literature is well established on the role sucrose and age play in a biofilms microbiology and chemistry, much less is known on how these factors influence the mesoscale morphology and mechanical properties. It was found that increasing sucrose concentration increased biofilm deposition and significantly reduced Young’s modulus. It also increased the adhesion of oral biofilms, while increasing age was shown to decrease adhesion only. This was associated with increasing EPS to bacteria ratios due to sucrose and decreasing this ratio due to aging. This analysis approach is not restricted to the type of biofilm under investigation, nor the *in-vitro* conditions in which future researchers wish to investigate. This approach can now be used in future to elucidate the effect of potential removal strategies.
